# Germ Busters: Environmental Cleaning in the Prevention of CLABSIs in Pediatric oncology patients”

**DOI:** 10.1097/pq9.0000000000000273

**Published:** 2020-04-22

**Authors:** Stephanie C. Gehle, Jessica Howard, A. Brooke Criddle, Corinne Corrigan, Elizabeth Mack

**Affiliations:** From the Medical University of South Carolina.

**Keywords:** central line-associated bloodstream infection, electronic health record, mucosal barrier infection, hospital-acquired condition, Medical University of South Carolina

## Background:

Pediatric hematology/oncology patients are at high risk for CLABSIs due to immunosuppression, high device utilization, and mucosal barrier injury related to chemotherapy.

## Objectives:

We aim to achieve 90% compliance with adjunct CLABSI prevention bundles (daily care, oral care, environmental care) in the pediatric hematology/oncology population to reduce CLABSI.

## Methods:

We began a series of plan-do-study-act cycles aimed at improving compliance with the adjunct CLABSI bundles for pediatric oncology patients. In October 2016, our quality team began monthly meetings with bedside nurse champions, oncologists, environmental services, infection prevention, and nurse techs to identify system barriers. In May 2017, we implemented oral care and daily care bundles on the unit, including toothbrushing, mouthwash, lip balm, daily chlorhexidine treatments, linen, and clothing change. In February 2018, an order set including adjunct bundles was implemented. In summer 2018, we conducted daily real-time audits and provided the team with feedback to improve compliance. We began auditing routine and terminal room cleaning using GloGerm on high-touch surfaces to share data feedback with environmental services. In November 2018, our oncologist implemented levofloxacin prophylaxis. In December 2018, we implemented prompts in the electronic health record for each adjunct bundle element. In June 2019, we began to track terminal cleans and 30-day room changes in the EHR and alert charge nurses when patients are approaching the 30-day limit so they can be moved to a cleanroom.

## Results:

**Fig. 1.**
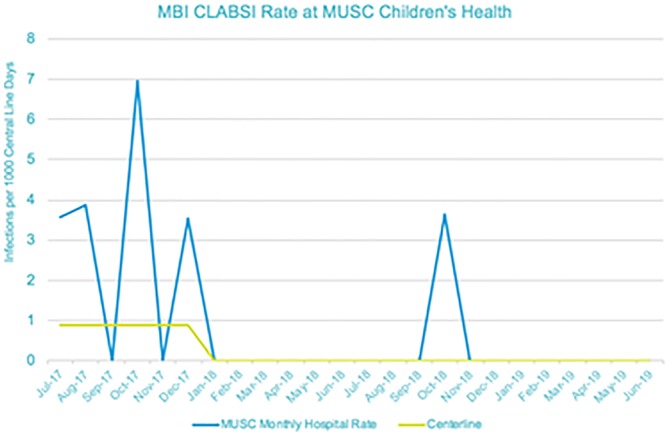
MBI CLABSI Centerline Shift at MUSC, July 2017–June 2019.

**Fig. 2.**
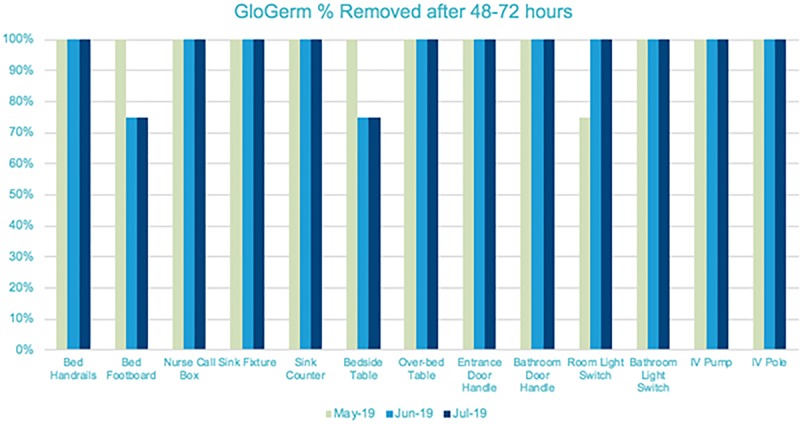
GloGerm Audits on High Touch Cleaning, May–July 2019.

**Fig. 3.**
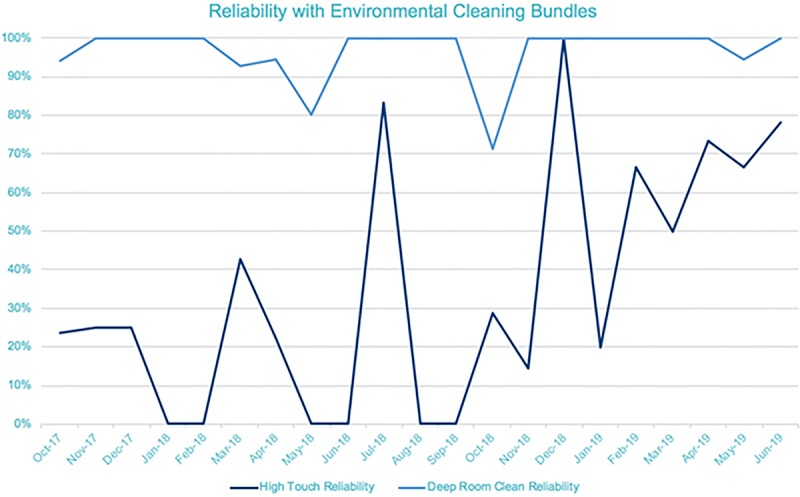
Reliability of High Touch Cleaning and Deep Room Cleaning, 2017–2019.

We have had 1 MBI CLABSI in January 1, 2018–May 31, 2019 (Fig. 1). Compliance with deep clean and high touch surfaces has improved with HAC documentation and robust team feedback using GloGerm and EHR monitoring of room changes for terminal cleans (Figs. 2 and 3).

## Conclusions:

We demonstrate the preventability of MBI CLABSIs in this immunocompromised population concurrent with an increase in environmental care bundle compliance.

